# Exacerbation of Anti‐Cytomegalovirus Immunity and Mobilization of γ9δ1 T Cells During the Acute Phase of Hepatitis E Virus Infection

**DOI:** 10.1111/1348-0421.70034

**Published:** 2025-12-15

**Authors:** Marion Retailleau, Fanny Martini, Florence Abravanel, Nassim Kamar, Jacques Izopet, Eric Champagne

**Affiliations:** ^1^ Centre National de la Recherche Scientifique (CNRS), Institut National de la Santé et de la Recherche Médicale (INSERM), UMR1291—CNRS UMR5051, Infinity Université de Toulouse Toulouse France; ^2^ CHU Toulouse, Hôpital Purpan, Laboratoire de virologie, National Reference Centre for Hepatitis E Toulouse France; ^3^ CHU Toulouse, Hôpital Rangueil, Service de Néphrologie et Transplantation Toulouse France

**Keywords:** cytomegalovirus, gamma delta T cells, hepatitis E virus, innate immunity, organ transplantation

## Abstract

HEV causes chronic infections that are detrimental to immunocompromised patients. Previous studies showed alterations of γδ T cell subsets at the acute phase of HEV infection. To assess a possible role of CMV, we have examined the frequencies and responses to CMV and HEV of blood γδ T cell subsets from control donors and acute‐phase HEV patients with or without CMV. CMV DNA was mostly undetectable in the blood of CMV‐seropositive HEV patients, and anti‐CMV antibodies were only slightly elevated. However, Vγ9^neg^Vδ1^pos^ cells were enriched in vivo, suggesting an increased CMV burden. In contrast, γ9δ1 cells were depleted in most HEV patients, regardless of CMV status. Culturing with IL‐2 and IL‐15 led to strong γ9δ1 T cell enrichment in samples from HEV patients. After IL‐2/IL‐15 sensitization, analysis of IFN‐γ responses to CMV‐infected fibroblasts or hepatocarcinoma cells (with IL‐18) showed innate responsiveness to CMV in γ9δ1, γ9δ2, and γ9^neg^δ1^pos^ cells in some control subjects and CMV‐seronegative patients. However, these responses were selectively exacerbated in CMV‐seropositive HEV patients, who also showed significant αβ T cell responses to CMV. This indicates reactivation of anti‐CMV immunity. Responses to HEV‐infected HepG2 cells remained undetected. IFN‐γ responses were not associated with TCR downmodulation in γ9δ2 or γ9^neg^δ1^pos^ cells. However, IFN‐γ‐producing γ9δ1 from HEV patients were characterized by high TCR expression, which was downmodulated after stimulation with CMV‐infected fibroblasts or CMV/HEV‐coinfected HepG2 cells. We conclude that γ9δ1 T cells are selectively mobilized in HEV patients and can be activated by CMV.

**Abbreviations**
ATCC: American Type Culture Collection
CMV: cytomegalovirus
EphA2: ephrin type A receptor 2
ffu: fluorescence focus forming unit
gc: genome copy
HEV: hepatitis E virus
LCM: lymphocyte culture medium
mfi: mean fluorescence intensity
PBMC: peripheral blood mononuclear cells
PFA: paraformaldehyde
TCR: T cell receptor

## 1 Introduction

Hepatitis E virus (HEV) is a single‐stranded positive RNA virus and the most common agent of acute hepatitis worldwide. Four genotypes frequently infect humans. In industrialized countries, HEV3 and HEV4 infections are prevalent and typically result in self‐limiting hepatitis. However, in immunocompromised individuals, particularly in the context of organ transplantation, infections become chronic in about two‐thirds of individuals and may progress to cirrhosis [1]. This progression can be prevented or treated with ribavirin therapy, though the virus persists in some patients despite treatment [2]. Better characterizing the natural immune response after HEV infection is important for understanding the pleomorphic evolution of the disease and viral persistence.

Regarding HEV3 infections [3], we have focused our attention on γδ T cells. This quantitatively minor population of T lymphocytes contributes to tissue homeostasis, anti‐cancer immunity, and responses to viral, bacterial, and parasitic infections [4, 5, 6, 7]. Subsets of γδ T cells are often defined by the V‐gene families that comprise their TCR. In humans, the Vγ9^pos^Vδ2^pos^ subset usually predominates in the blood of healthy individuals. This subset is mobilized during bacterial or parasitic infections due to its ability to detect cells producing exogenous phosphoantigens. It may also be activated or altered during viral infections through TCR‐dependent detection of phosphoantigenic metabolites [6, 8, 9] or TCR‐independent mechanisms. Currently, it is accepted that this subset displays innate reactivity, whereas other subsets collectively forming a “non‐Vγ9Vδ2” population have more adaptive potential [10, 11, 12]. Non‐Vγ9Vδ2 T cells predominate in tissues [10] and are subject to in vivo oligoclonal amplifications in the course of acute CMV infections. In the context of organ transplantation, their amplification correlates with CMV clearance [13, 14]. Recently, an expansion of cells with a Vγ9^neg^Vδ2^pos^ phenotype was found to indicate severe CMV disease in kidney transplant patients [15]. After infection, latent CMV persists lifelong in cells of the myeloid lineage and CD34+ precursors. It may reactivate in infectious or inflammatory contexts [16, 17, 18, 19, 20]. Carriage of CMV is responsible for significant biases in the T cell repertoires in the elderly, resulting in an accumulation of CMV‐specific CD8+ T cells as well as non‐γ9δ2 T cells with terminally differentiated phenotype [14, 21, 22, 23, 24]. The accumulation of effector memory Vδ2^neg^ T cells with age in healthy CMV carriers has been shown to correlate with an increase in anti‐CMV antibodies and may thus reflect CMV burden [25].

Alterations in γδ T cell subsets have been observed in various viral infections, including HIV, HBV, HCV, and dengue and influenza viruses. However, the factors that determine γδ T cell responses remain unclear. Coinfections with highly prevalent and persistent viruses, such as Herpes family viruses, are potential confounding factors, making viral targets for these responses elusive [26]. The current view is that γδ T cells target stress‐induced cellular determinants. Such targets have been characterized in the case of anti‐CMV responses and include molecules related to and unrelated to MHC [27]. However, responses appeared to be specific to CMV, as the activated γδ subsets did not recognize fibroblasts infected with herpes viruses other than CMV. In addition, cross‐reactivity against leukemic or intestinal cancer cells is well documented [28].

In previous studies, we have observed alterations of γδ T cell subsets during the acute phase of HEV infection in both immunocompromised transplant patients and immunocompetent individuals [29, 30]. Interestingly, in vivo amplification of cells with a non‐γ9δ2 phenotype was only observed in CMV‐seropositive (CMV^pos^) patients, despite the absence of detectable CMV reactivation based on CMV genome detection in serum or plasma. Although contact with HEV‐infected cells promoted IL‐10 secretion by γδ cells, attempts to expand putative HEV‐specific γδ T cells from patients have failed [30]. Therefore, it was unclear whether the γδ T cell amplifications were truly HEV‐specific. This study aimed to reexamine γδ T cell subsets in HEV patients and assess their responses to HEV and CMV in vitro compared to healthy individuals.

## 2 Materials and Methods

### 2.1 Cell Samples From HEV Patients and Healthy Control Donors

PBMC and serum samples collected from HEV3‐infected patients during the first 2 weeks of clinical or biological symptoms were obtained from the DC‐2015‐2450 collection, which is hosted by the Biological Resources Center of the Toulouse University Hospital. PBMC and plasma from healthy adults were provided by the French National Blood Service and processed and stored by the local Immunomonitoring platform registered with the Ministry of Higher Education and Research (DC‐2016‐2772). Patients' consent or non‐opposition to the use of samples for HEV research was registered according to French ethical guidelines. The study was approved by the French South‐West & Overseas Ethics Committee. Experiments were performed in accordance with the Declaration of Helsinki guidelines. Patients' characteristics are provided in the Supporting Information.

Serological (anti‐HEV IgM and IgG) tests were performed on blood sera or plasma using Wantai enzyme immunoassays (Wantai Biological Pharmacy Enterprise, Beijing, China) following the manufacturer's recommendations. Acute HEV infection was diagnosed in patients by positivity for anti‐HEV IgM, elevated ALT, and/or the presence of the HEV RNA in the blood by quantitative RT‐PCR (RealStar HEV RNA kit 2.0, Altona Diagnostics). Anti‐cytomegalovirus viral lysate antibodies were titrated using the CMV IgG Abbott diagnostic test (Abbott Laboratories). CMV viremia was determined by PCR as previously reported, with a sensitivity of 300 IU/mL [31].

### 2.2 Cells Lines and Viral Isolates

The MRC‐5 fibroblasts and the HepG2/C3A hepatocarcinoma cells were obtained from the American Type Culture Collection (ATCC) and cultured in DMEM supplemented with 10% FCS, penicillin, and streptomycin (culture medium).

The CMV strain AD169 (CCL‐171) was also obtained from ATCC. The virus was amplified on MRC‐5 fibroblasts and collected from supernatants by ultracentrifugation at 70,000 g for 40 min. The virus was then resuspended in culture medium and frozen in aliquots. The CMV titer in the viral stock and the supernatant from infected cultures was determined by infection of MRC‐5 monolayers with serial dilutions. Then, the fibroblast layers were immunostained with anti‐CMV‐IE Ab on Day 4, and the fluorescence focus forming unit (ffu) counts were determined by immunofluorescence microscopy.

HEV infections were performed using the clinical isolate HEV‐3f (TLS09‐0) [32]. The virus was propagated in a MAVS‐knockdown HepG2 cells (a gift from Z. Feng, Ohio State University) [33] and purified from pooled cell lysates and supernatants by ultracentrifugation for 1 h at 70,000 g. The virus was then resuspended in PBS and kept frozen in aliquots at −80°C. Concentrations of HEV RNA in viral stocks were determined using a one‐step RT‐qPCR (Taqman) targeting the overlapping region of ORF2 and ORF3, as previously described [34]. A viral stock titrating 2 × 10^12^ genome copies (gc) per mL was used for experiments. To quantify HEV RNA in infected HepG2 cells, the cell layers were washed with PBS and collected in RNA lysis buffer by scraping, and the RNA was extracted using the Quick‐RNA miniprep kit (Zymo Research). Reverse transcription was carried out on 1 µg of RNA using a Sensifast cDNA synthesis kit (Meridian Bioscience). PCR amplification was performed in duplicate using 1/20 of the first‐strand reaction, Cybergreen mix (Roche Diagnostics), and ORF2/3 region‐specific primers (0.5 µM each). The LightCycler 480 and software from Roche Diagnostics were used for fluorescence detection of PCR products and threshold cycle (Ct) determination. GAPDH cDNA amplification was used as a reference to calculate the ΔCt (Ct gene − Ct GAPDH). Results from duplicate amplifications were averaged. Primers sequences are listed in the Supporting Information.

### 2.3 Lymphocyte Cultures

After thawing, the PBMC samples were cultivated at a concentration of 5 × 10^6^ cells/mL, for a 7‐day primary culture in lymphocyte culture medium (LCM: RPMI‐GlutaMAX [LifeTechnologies] supplemented with 10% FCS, penicillin, streptomycin, and sodium pyruvate) in the presence of IL‐2 (50 ng/mL) and IL‐15 (20 ng/mL), with half of the medium changed on Day 4.

For co‐cultures with cytokine‐sensitized PBMC, MRC5 and HepG2 cells were plated in 24‐well plates 3 days before infection (2.5 × 10^4^ and 10^5^ cells/cm^2^, respectively) to reach ~80% confluence on the day of infection. The cells were then infected with CMV at a multiplicity of infection of 0.3 ffu/cell, HEV (200 gc/cell), or both viruses. After 24 h, the infected and noninfected adherent cells were washed twice with DMEM. To assess IFN‐γ production and TCR modulation, the sensitized PBMC were washed and incubated for 4 h in LCM containing IL‐2 (20 ng/mL) and IL‐18 (10 ng/mL). Then the PBMC were added to MRC‐5/HepG2 cells (1.5 × 10^6^ cells/well) in a final volume of 1.5 mL, along with IL‐2 (20 ng/mL) and IL‐18 (10 ng/mL). Parallel control wells were coated with anti‐CD3 (MEM57 clone from ExBio Praha, 16 h, 5 µg/mL in PBS) and washed with RPMI for control TCR stimulation. After a 16‐h co‐culture with no cytokine secretion blocker, non‐adherent cells were recovered from all wells and stained as described below.

IL‐2, IL‐15, and IL‐18 were premium‐grade cytokines purchased from Miltenyi Biotech.

### 2.4 Flow Cytometry Analysis

For the analysis of lymphocyte subsets, cells from PBMC samples or cultured cells were washed once in PBS and stained with Live Dead Yellow (Thermo Fisher Scientific, 20 min, 0.5 µM) to identify dead cells. Then, the cells were washed with PBS and suspended in PBS plus 10% human serum for 15 min to saturate the Fc receptors. Surface staining was performed in staining buffer (PBS, 5% FCS, and 0.02% NaN_3_). Anti‐pan‐γδ was added first on pelleted cells for 20 min, followed by the addition of a mix of anti‐CD3, anti‐Vδ2, anti‐Vδ1, and anti‐Vγ9 for 40 min. After washing, the cells were fixed with PBS plus 2% paraformaldehyde (PFA) for 15 min at 4°C. When intracellular staining was performed, a fixation and permeabilization kit for cytokine detection (Invitrogen) was used according to the manufacturer's recommendations. Anti‐IFN‐γ was then added for 45 min in permeabilization buffer. After washing, cells were fixed again for 10 min in 2% PFA. Fluorescence was acquired using a Fortessa X20 cytofluorometer (Becton Dickinson). Antibody details are provided in the Supporting Information.

### 2.5 Data Treatment and Statistical Analysis

To analyze TCR modulation on T cells after lymphocyte culture, we collected the mean fluorescence intensities (mfi) of the pan‐γδ‐TCR staining on IFN‐γ^Low^ and IFN‐γ^Hi^ cells for each subset. The TCR mfi on IFN‐γ^Low^ cells in noninfected cultures was averaged for each patient/control group, and the resulting value was used for normalization of the corresponding dataset.

Nonparametric statistics were used in Section 3 unless specified in the figure legends. Statistics were calculated using the GraphPad Prism software.

## 3 Results

### 3.1 Sample Individuals and Experimental Design

**Figure 1:**
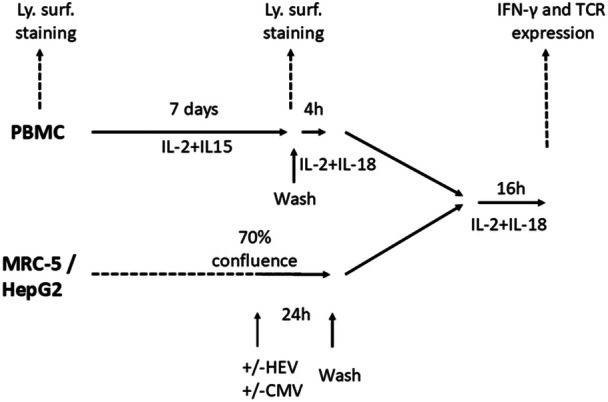
Experimental strategy. PBMC samples were examined by flow cytometry after thawing (ex vivo) and after 7 days of amplification in IL‐2+1L‐15. Viable cells were then transferred to medium containing IL‐2+IL‐18 and seeded on wells with MRC5 or HepG2 cell layers, previously infected or not. Flow cytometry was then used to monitor intracellular IFN‐γ accumulation in lymphocyte subsets and the level of TCR expression.

Twelve patients in the acute phase of HEV infection, including six organ‐transplant patients, were characterized for their CMV seropositivity and CMV‐DNAemia. Among the six CMV‐seropositive (CMV^pos^) patients, one had detectable CMV‐DNA in the serum. A control panel of 16 healthy donors, characterized for their HEV and CMV seropositivity, was constituted, including an equal number of HEV‐seropositive individuals and HEV‐seronegative donors (Table 1 and Supporting Information). CMV^pos^ individuals were slightly older (*p* = 0.10, Mann–Whitney). The experimental strategy is depicted in Figure 1 and explained further in this section.

**Table 1:** Control and patient groups.

	Cont‐CMV^neg^	Cont‐CMV^pos^	HEV‐CMV^neg^	HEV‐CMV^pos^
N (SOT)^a^	8 (0)	8 (0)	5 (2)	7 (4)
Gender M/F	5/3	3/5	3/2	4/3
Age (range)^b^	40 (26–63)	49.9 (30–67)	43.4 (22–77)	53 (24–73)
HEV‐IgG pos/neg	4/4	4/4	5/0	7/0
CMV IgG titer (range)^c^	Neg. (0–0.8)	306 (28–1114)	Neg. (0–0.5)	1316 (120–5890)

^a^ Solid organ transplant.

^b^ *p* > 0.9 for all group combinations (Kruskal–Wallis test).

^c^ Log IU; titers < 1 are considered negative; comparison Cont‐CMV^pos^/HEV‐CMV^pos^: *p* = 0.094 (Mann–Whitney test).

### 3.2 Quantification of γδ T Cell Subsets Ex Vivo

PBMC from control and patient samples were phenotyped to quantify γδ T cell subsets ex vivo by flow cytometry. Fluorescence co‐staining of lymphocytes for CD3, γδ‐TCR, Vδ1, Vδ2, and Vγ9 can identify six γδ T cell subsets: γ9δ1, γ9δ2, γ9δX (Vγ9^pos^Vδ1^neg^Vδ2^neg^), γXδ1 (Vγ9^neg^Vδ1^pos^Vδ2^neg^), γXδ2 (Vγ9^neg^Vδ1^neg^Vδ2^pos^), and γXδX (Vγ9^neg^Vδ1^neg^Vδ2^neg^). However, γ9δX, γXδ2, and γXδX cells were rare or undetectable in some conditions or individuals. Gating strategies for flow cytometry analysis and representative histograms are shown in the Supporting Information.

**Figure 2:**
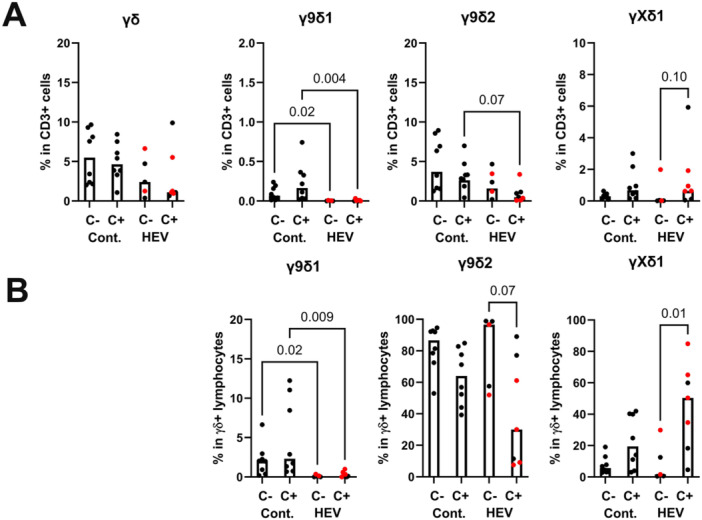
γδ T cell subsets ex vivo and after culture with IL‐2+IL‐15. The frequencies of γδ T cell subsets ex vivo were determined by flow cytometry on PBMC samples. (A) Relative frequency of γδ T cells and major subsets among CD3 cells. (B) Frequencies of major subsets among total γδ T cells. Controls and patients are separated according to their seropositivity for CMV (C−/C+). Bars indicate median values. Red dots represent transplanted HEV patients. Statistical analysis by Kruskal–Wallis test (*n* = 8/8/5/7). Only *p* values < 0.1 are indicated.

Ex vivo, γ9δ2 cells dominated non‐Vγ9Vδ2 γδ T cells similarly in most CMV^pos^ and CMV^neg^ control individuals. This was also true for CMV^neg^ HEV patients. In contrast, CMV^pos^ HEV patients had increased percentages of γXδ1 T cells in their γδ T cells. γXδ1 T cells increased proportionally among CD3+ cells, suggesting a moderate expansion of this subset in peripheral blood. Thus, HEV infection in CMV carriers is associated with alterations described in open CMV infections or CMV reactivation [14, 35, 36, 37]. Notably, γ9δ1 cells were consistently rare in CMV^pos^ and CMV^neg^ HEV individuals ex vivo, whereas they represented a significant subpopulation of the γδ T cells in controls (Figure 2).

### 3.3 Anti‐CMV IgG Titers in Seropositive Individuals

**Figure 3:**
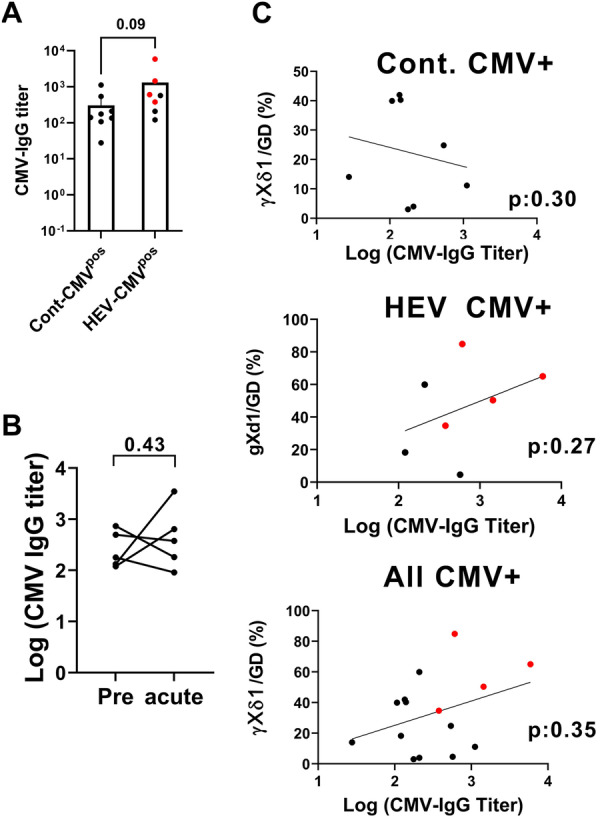
Anti‐CMV IgG titers in CMV^pos^ controls and HEV patients. CMV‐IgG antibody levels were determined in sera from CMV‐seropositive control individuals and HEV patients. Red dots represent transplanted patients. (A) Comparison of CMV‐IgG titers between CMV‐seropositive control and HEV samples. (B) Comparison of titers before and during HEV‐acute infection in five transplanted patients (Mann–Whitney test). (A) Bars indicate means; (A, B) statistics: Mann–Whitney test. (C) Simple linear regression and correlation analysis of CMV‐IgG titers and γXδ1 frequency in CMV^pos^ controls and patients (*p* value of Spearman's correlation).

Although CMV DNA was detected in one CMV^pos^ HEV patient, the observation of γδ T cell subset modifications, such as the gXd1 bias, in most CMV^pos^ patients (Figure 2) could reflect occult CMV reactivation. We hypothesized that this might also result in an elevation of anti‐CMV antibodies. Thus, anti‐CMV IgG titers were analyzed in CMV^pos^ individuals (Figure 3). Anti‐CMV IgG titers in serum were slightly higher in CMV^pos^ HEV patients than in CMV^pos^ controls. Serum samples from before the acute phase were available for five HEV patients who had undergone solid organ transplantation. No consistent elevation of anti‐CMV IgG was observed in these patients. Additionally, anti‐CMV IgG levels did not significantly correlate with the frequency of γXδ1 cells in either controls or patients. Thus, the CMV serological data suggest an increased CMV burden in HEV patients, but do not support systemic CMV replication.

### 3.4 Quantification of γδ T Cell Subsets After Expansion With Cytokines

**Figure 4:**
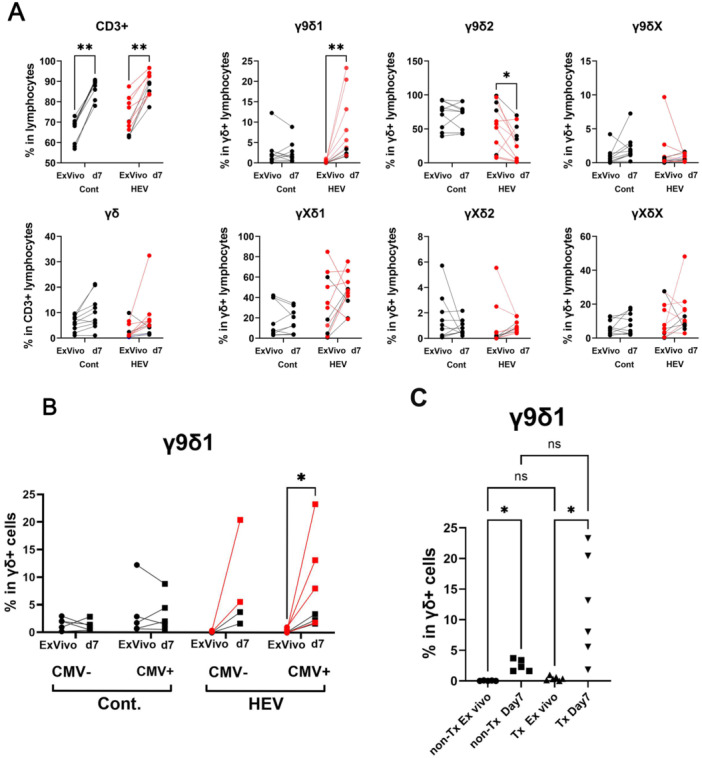
Expansion of γδ T cell subsets in the presence of IL‐2 and IL‐15. The frequencies of γδ T cell subsets were determined by flow cytometry after 7 days of PBMC culture in the presence of IL‐2 and IL‐15 and compared to ex vivo frequencies in controls and HEV patients. (A) Frequency of total CD3+ among lymphocytes, γδ T cells among CD3+ cells, and γδ T cell subsets among γδ cells. (B) Comparison of γ9δ1 T cell frequencies in controls and patients separated according to their CMV status. (C) Comparison of γ9δ1 T cell frequencies in immunocompetent (non‐Tx) and transplanted (Tx) HEV patients. Statistics (A, B, C): Kruskal–Wallis test, *p > 0.05; **p < 0.01; ns: *p* > 0.05.

Due to the limitation in the amount of biological material available for evaluating antiviral reactivity, we aimed to amplify γδ T cells in vitro with cytokines without additional antigenic stimulation to favor amplification of T cells that are sensitized in vivo. The combination of IL‐2 + IL‐15 is reported to promote amplification of activated γδ T cells while preserving viability [15, 38, 39, 40]. Thus, PBMC from patients and controls were cultivated for 7 days in a medium supplemented with IL‐2 and IL‐15. T cell subsets quantification was assessed again by flow cytometry to evaluate the effect of culture on the relative proportions of γδ subsets (Figure 4). The total number of viable cells recovered increased slightly compared to the initial number (by a factor of 1.2–1.5; not shown). Nevertheless, the culture led to an enrichment of CD3+ cells. No significant enrichment of γδ over αβ cells occurred in patients or controls. Among the γδ T cells, the proportions of the γ9δ2, γ9δ1, and γXδ1 subsets were not consistently altered by the culture with cytokines in the controls. In contrast, in CMV^neg^ and CMV^pos^ HEV patients, the relative proportion of γ9δ2 cells was decreased, while a substantial population of γ9δ1 cells emerged (Figure 4B). This increase was more pronounced in cultures from transplanted patients, though the patient sample size was insufficient for statistical significance (Figure 4C). Thus, although γ9δ1 T cells are depleted ex vivo, they appear highly proliferative in the presence of IL‐2 and IL‐15 in HEV patients.

### 3.5 IFN‐γ Response of T Cell Subsets to Infected Target Cells

**Figure 5:**
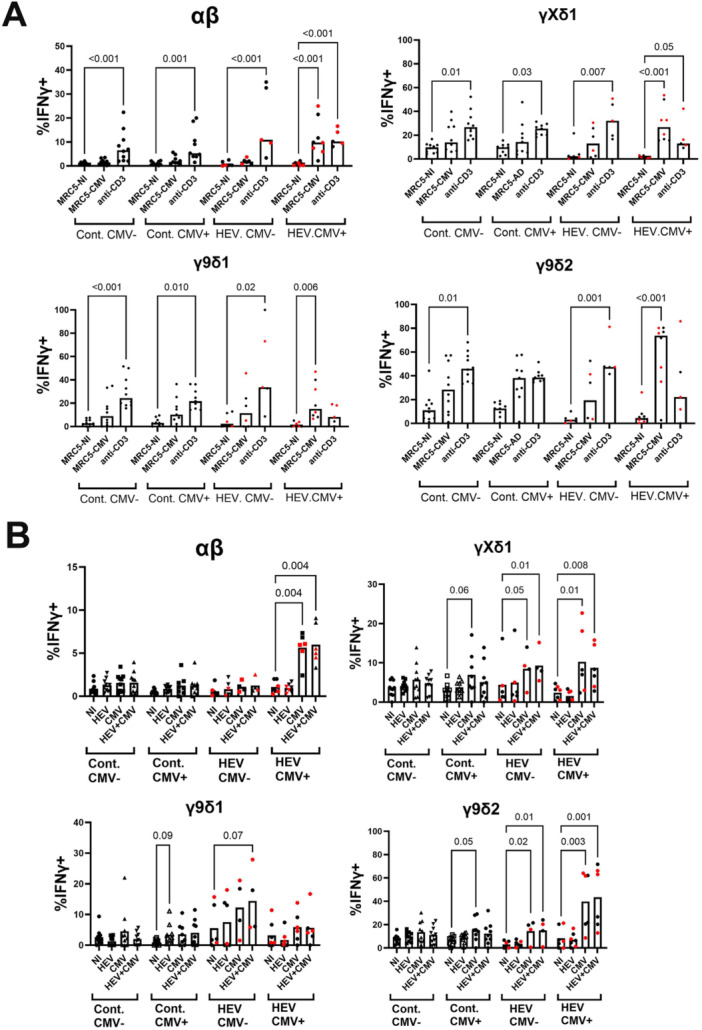
IFN‐γ response and TCR modulation of γδ T cells stimulated with CMV/HEV‐infected cells. PBMCs from controls (Cont.) and HEV patients were primed for 7 days with IL‐2 and IL‐15 and stimulated for 16 h with (A) MRC‐5 or (B) HepG2 cells, either noninfected (NI), pre‐infected for 24 h with CMV (A, B) or HEV (B), or co‐infected (B). Stimulation with plastic‐coated anti‐CD3 was used as a positive control. HEV patients are separated according to their CMV seropositivity (CMV−/CMV+). IFN‐γ responses were determined by intracellular cytokine staining. The results are expressed as the percentage of IFN‐γ^pos^ cells in the indicated T cell subsets. Bars indicate median values. Red dots represent transplanted HEV patients. Statistical analysis by Kruskal–Wallis test (*n* = 10/9/6/8).

We then examined whether the expanded cells reacted to CMV‐ and/or HEV‐infected targets. Intracellular IFN‐γ induction was chosen as a readout, as this cytokine is usually produced or coproduced in human γδ T cells in response to viral infection. To monitor T cell subset reactivity to CMV, we used CMV‐permissive MRC‐5 cells for stimulation. We used HepG2 hepatocarcinoma cells, which are permissive for CMV and HEV, to monitor T cell reactivity to target cells infected with HEV, CMV, or both (see Section 3 and [30, 41, 42]). The cells were infected for 24 h and co‐cultured with cytokine‐sensitized lymphocytes in the presence of IL‐2 and IL‐18. This cytokine combination was used because preliminary testing indicated that it allows optimal IFN‐γ induction without the background response of the synergistic IL‐12/IL‐18 combination [43, 44, 45]. Then, intracellular IFN‐γ accumulation was measured by flow cytometry in T cell subsets. TCR stimulation with plastic‐coated anti‐CD3 was used to assess the ability of subsets to produce IFN‐γ in the presence of the same cytokines (Figure 5). Under these conditions, there was no significant background IFN‐γ induction in the absence of infected cells or anti‐CD3 stimulation.

For most control donors, stimulation by CMV‐infected MRC‐5 fibroblasts induced IFN‐γ production in cytokine‐sensitized total γδ T cells. This response was more significant in CMV^pos^ donors, presumably due to in vivo clonal selection. However, this response was not statistically significant when γδ subsets were considered separately. This suggests that γ9δ2, γ9δ1, and γXδ1 subsets were not simultaneously involved in the response of controls. αβ (CD3^pos^pan‐γδ^neg^) T cell responses were not detected in controls.

γδ‐T cells from most HEV patients reacted to CMV‐infected MRC‐5 cells, and CMV^pos^ HEV patients had highly significant γδ T cell responses. Stimulation of IFN‐γ production by CMV was statistically significant in the three major subsets in this group of patients. Notably, the IFN‐γ response of γδ T cells to anti‐CD3 stimulation was low in CMV^pos^ HEV patients, suggesting that Th1 differentiation is lower in this group. Most importantly, CMV^pos^ HEV patients had a high frequency of αβ T cells that responded to CMV‐infected MRC‐5 cells (Figure 5A).

IFN‐γ production after stimulation with CMV‐infected HepG2 cells was never significant in the control group, though weak responses were detected in CMV^pos^ controls. CMV‐infected HepG2 cells most significantly induced IFN‐γ expression in αβ and γδ T cells from CMV^pos^ HEV patients. However, this induction was significant in γ9δ2 and γXδ1 cells but not in γ9δ1 cells. HEV alone or in coinfection with CMV did not influence IFN‐γ production in γ9δ2, γ9δ1, or γXδ1 T cells in controls or patients (Figure 5B).

Thus, the IFN‐γ response to CMV‐infected cells in this setting is an innate property of a large fraction of γ9δ2 and non‐γ9δ2 cells. However, their ability to respond increases during the acute phase of HEV infection in patients carrying CMV, which coincides with the activation of conventional T cell immunity to CMV. In this setting, HEV infection of target cells does not promote IFN‐γ production in lymphocytes.

### 3.6 TCR Modulation After Stimulation With Infected Targets

**Figure 6:**
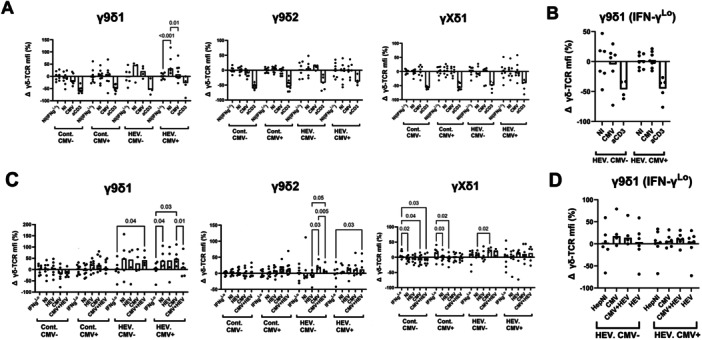
TCR modulation of γδ T cells stimulated with CMV/HEV‐infected cells. PBMCs from controls (Cont.) and HEV patients were primed and stimulated as described in Figure 5. (A, C) The mean fluorescence intensities (mfi) of pan‐γδ TCR staining were recorded in IFN‐γ^Pos^ and IFN‐γ^Low^ cells for each subset (pan‐γδ staining). Results are expressed as the percentage of variation of TCR expression relative to IFN‐γ^Low^ cells (pan‐γδ staining) for each subset: relative TCR expression after stimulation with (A) uninfected or CMV‐infected MRC5 fibroblasts and (C) HepG2 cells infected with CMV, HEV, or both viruses. (B, D) Relative TCR expression on IFN‐γ^Low^ cells after stimulation with (B) infected MRC5 and (D) HepG2 cells, using cocultures with uninfected cells as a reference. Data normalization was applied and is described in Section 2. Statistical analysis used a two‐way ANOVA mixed effects model.

The increased IFN‐γ response of lymphocyte subsets in the presence of CMV‐infected cells may result from TCR cross‐linking or co‐stimulatory signals, such as NK receptor or TLR stimulation. As TCR stimulation induces TCR modulation on the cell surface, TCR levels were recorded. This was performed on IFN‐γ^Hi^ and IFN‐γ^Lo^ cells for each subset (Figure 6). TCR cross‐linking with anti‐CD3 induced significant TCR downmodulation in all γδ subsets. However, TCR downmodulation was not detected in γ9δ2 and γXδ1 T cells following stimulation with infected fibroblasts. This suggests that IFN‐γ production by these T cells is, in large part, TCR‐independent in this setting. Unlike other subsets, IFN‐γ‐producing γ9δ1 T cells from HEV patients exhibited high TCR expression in uninfected cultures, which significantly decreased upon stimulation with CMV‐infected fibroblasts. Thus, this subset appears to be particularly CMV‐reactive through a process that may involve the TCR (Figure 6A). No similar downmodulation was detected in IFN‐γ^Lo^ cells upon stimulation with CMV‐infected MRC5 cells; therefore, virus‐responsive cells producing another cytokine appear to be rare (Figure 6B).

No TCR downmodulation was observed with CMV or HEV in single infections of HepG2 cells. Nevertheless, γ9δ1 T cells from CMV^pos^ HEV patients displayed higher TCR expression, which was downmodulated on IFN‐γ‐producing cells upon exposure to HEV + CMV co‐infected HepG2 cells (Figure 6C). Thus, although infected HepG2 cells did not significantly increase the frequency of γ9δ1 cells producing IFN‐γ, the data suggest that TCR triggering of this subset occurs in CMV^pos^ HEV patients due to the synergistic effect of CMV and HEV. Similar to MRC‐5 cocultures, TCR downmodulation was not detected in the IFN‐γ^Lo^ cells (Figure 6D). Overall, these results suggest that TCR stimulation occurs in the γ9δ1 cells of CMV^pos^ HEV patients after triggering by virus‐infected cells. This is most evident after stimulation with CMV‐infected cells.

### 3.7 Effect of Coinfection on CMV and HEV Replication in HepG2 Cells

**Figure 7:**
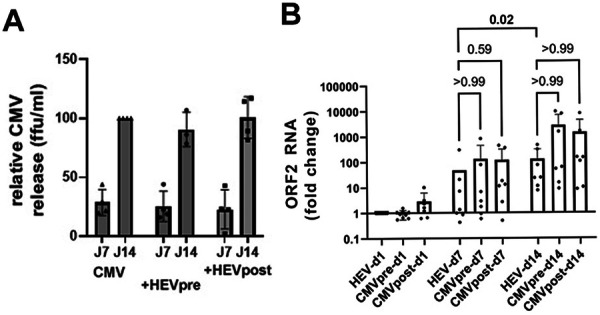
HEV and CMV_AD169_ replication in MRC‐5 and HepG2 cells. (A) Determination of infectious CMV release in day‐7 and day‐14 supernatants of HepG2 cells in single CMV infections or coinfections with HEV, added either 24 h before (HEVpre) or after CMV (HEVpost). (B) RT‐PCR quantification of HEV genome in HepG2 lysates 1, 7, or 14 days after HEV infection, alone or in coinfection with CMV, added either 24 h before (CMVpre) or after HEV (CMVpost) (*n* = 6 independent experiments). Statistical analysis by Friedman's ANOVA; (A) *n* = 4; (B) *n* = 6.

As HEV and CMV synergize in vivo and possibly in vitro to activate γδ T cells, we tested whether coinfection alters HEV or CMV replication (Figure 7). After CMV infection in HepG2 cells, infectious CMV particles accumulated in the cell culture medium over 14 days, a process that was not significantly affected by HEV coinfection when HEV was added 24 h before or after CMV infection (Figure 7A). HEV infection of HepG2 cells resulted in progressive HEV RNA increase in cell lysates over 14 days. This was not significantly affected by CMV added either 24 h before or after HEV (Figure 7B). Thus, the synergistic effects of the two viruses do not simply result from increased replication following coinfection. Additionally, HEV's slow replication might limit the detection of responses against HEV in the experimental setting used in the above experiments.

## 4 Discussion

This study confirms that γδ T cells are mobilized during the acute phase of HEV infection and reveals specific alterations in individuals carrying CMV. The increase in γXδ1 and the moderate decrease in γ9δ2 cells in blood are consistent with previous reports [24, 46, 47] and support the idea that they are induced by a CMV reactivation, which remains generally occult. This study also reveals a shift in the behavior of γ9δ1 cells linked to HEV infection in vivo.

IFN‐γ responses against CMV‐infected cells are exacerbated in cells of CMV^pos^ HEV patients, including αβ and γδ T cell subsets. This most likely results from oligoclonal or polyclonal amplification [36] while subsets engage in a CMV response, supporting the notion that anti‐CMV immunity reactivates in most CMV carriers during the acute phase of HEV infection. CMV viremia was rarely detected in these patients, and CMV‐specific IgG titers did not consistently increase. This may be because IgG titers are tightly linked to the level of viremia [48], or it may be due to the fact that the blood trafficking of CMV‐reactive conventional and nonconventional T cells precedes the elevation of antibody titers upon viral reactivation in tissues. In line with a possible occult reactivation, previous reports have documented CMV shedding in secretions rather than in blood in CMV carriers [49, 50, 51]. Thus, CMV is most likely responsible for the relative depletion of γ9δ2 and the inflation of γXδ1 observed ex vivo. The rarity of CMV detection in serum and its lack of association with clinical manifestations, such as CMV disease, suggest that CMV infection is well controlled in most patients in our study, including those undergoing immunosuppressive treatment.

In HEV patients, no reactivity to HEV was detected through IFN‐γ responses. This may be due to HEV's non‐cytopathic and slow replicative properties. The experimental setting involved a short‐term (24‐h) infection, which induces a strong cytopathic effect with CMV but little HEV replication. This may hinder the detection of HEV effects, and alternative approaches, such as the transfection of HEV RNA replicons, could be useful [52]. Although the hepatocarcinoma line used in our study is particularly permissive for HEV [30, 33], the usage of primary hepatocytes as targets should also be considered in future work. Note that a specific reactivity to chronically infected HepG2 cells was not detected in previous studies using chronically infected cells [30].

CMV stimulation increased IFN‐γ accumulation in several γδ T‐cell subsets in HEV CMV^pos^ patients. This reactivity was observed in IL‐2/IL‐15‐sensitized cells; thus, it may be favored by the cytokine environment. Furthermore, IL‐18 was included in the stimulation step and was necessary for IFN‐γ induction. Interestingly, γ9δ2 responses to CMV‐infected MRC5 were particularly pronounced and detectable in several control individuals, even in the absence of CMV carriage. Thus, reactivity to CMV‐infected cells is an intrinsic property of many γδ T cells, including γ9δ2, γ9δ1, and γXδ1 subsets, without stringent TCR selectivity. Furthermore, the absence of TCR modulation in most instances suggests that this reactivity is largely TCR‐independent. In contrast to our results, Kaminsky et al. reported selective non‐γ9δ2 responses, despite using a similar sensitization protocol [15]. The differences in virus isolates and target fibroblasts used in the assays may explain the discrepancy: the CMV_AD169_ isolate used in our experiments is particularly cytopathic in MRC5 cells, which may influence recognition of viral ligands by stimulatory or regulatory coreceptors. It is also possible that bystander cells contributed, since γδ cells were not sorted in our setting.

The quantitatively minor subset of γ9δ1 cells exhibits a particular behavior in HEV patients. In previous reports, γ9δ1 cells have been isolated from a patient with Lyme arthritis [53, 54, 55, 56] and from renal or lung transplant patients [18, 28]. Some γ9δ1 T cell clones have been found to become activated upon recognizing metabolic stress during bacterial infections [52, 53, 54, 55, 56] or in the context of CMV infection [18, 28]. These cells frequently cross‐react with tumors [28, 57, 58], and one clone was found to recognize the ephrin receptor A2 (EphA2) stress antigen in a TCR‐dependent manner [18]. In our experiments, this subset was usually detected ex vivo in control samples but was depleted from the periphery during the acute phase of HEV infection, suggesting homing and sequestration in tissues, independent of CMV. Their high proliferative potential in the presence of IL‐2 and IL‐15 suggests priming in vivo during HEV infection. If so, their activation by CMV‐infected cells in vitro may result from the recognition of a common stress antigen induced by CMV and HEV. In contrast, the poor recognition of HEV‐infected cells could result from low expression of this antigen or the lack of co‐stimulation by HEV‐infected cells.

γ9δ1 T cells may infiltrate tumors, display cytotoxic activity against tumor cells, and exhibit a Th1 phenotype [57]. In our setting, a relatively low percentage of cells in this subset produced IFN‐γ after restimulation. Since the strategy and cytokine combinations used here favor the detection of Th1 cells, future investigations should assess the ability of γ9δ1 cells to express or differentiate into alternative functional types, such as Th17 or regulatory cells, in the context of antiviral responses. This differentiation might also be influenced by the viral environment, and previous investigations indicated that HEV‐infected cells were able to promote IL‐10 production in γδ T cells [30]. Thus, at present, it is not known whether these cells are bystanders, cytotoxic effectors, or regulatory cells. This differentiation can differ with the anti‐tumor or antiviral context of activation, and our study suggests that, in the context of HEV infections, it may also differ whether CMV is present or not. For example, γ9δ1 cells from CMV^neg^ HEV patients were not significantly reactive to CMV‐infected targets. Thus, we suspect that CMV in vivo promotes clonal selection. Thus, it will be important to investigate whether this is beneficial or not to patients and if this influences the progression and persistence of HEV infection in immunocompromised individuals.

The number of patients in the present study was limited by the amount of cells required by our cellular approach. This limitation prevented us from linking CMV carriage and progression of HEV infection. Moreover, the study included transplant patients, and the real impact of transplantation and immunosuppression in vivo also deserves further attention. To overcome these limitations, further studies using single‐cell analyses on more samples will be necessary.

## Ethics Statement

The study on patients and healthy donor samples was approved by the French South‐West & Overseas Ethics Committee and was registered at the Ministry of Higher Education and Research (DC‐2016‐2772 and DC‐2016‐2772). Patients' consent or non‐opposition for use in HEV research was registered as required by French ethical guidelines. Experiments were performed in agreement with the guidelines of the Declaration of Helsinki.

## Conflicts of Interest

The authors declare no conflicts of interest.

## Supporting information

1) Characteristics of patients. 2) Antibodies. 3) PCR primers. 4) Supplemental figure 1. Gating Strategy for γδ T cell subset identification by flow cytometry. 5) Supplemental figure 2. IFN‐γ detection in lymphocyte subsets by flow cytometry.

## Data Availability

The data that support the findings of this study are available from the corresponding author upon reasonable request.
