# Supplementary material for: Exacerbation of Anti‐Cytomegalovirus Immunity and Mobilization of γ9δ1 T Cells During the Acute Phase of Hepatitis E Virus Infection
Source: Microbiol Immunol. 2025 Dec 15;70(2):113–24. doi: 10.1111/1348-0421.70034 (PMC12868941; doi:10.1111/1348-0421.70034)
Supplement: Supplementary file 1 — 1) Characteristics of patients. 2) Antibodies. 3) PCR primers. 4) Supplemental figure 1. Gating Strategy for γδ T cell subset identification by flow cytometry. 5) Supplemental figure 2. IFN‐γ detection in lymphocyte subsets by flow cytometry. [file MIM-70-113-s001.pdf]

SUPPLEMENTAL MATERIAL AND METHODS INFORMATION

1) Characteristics of patients

Clinical status	Immunosup.	VHE RNA (log UI/ml)	Génotype	Gender	age	CMV IgG titer
Hepatitis	None	7.3	3c	F	39	<0.4
Hepatitis	None	4.1	3c	F	40	0.4
neuro. symptoms	None	2.05	ND	M	39	<0.4
neuro. symptoms	None	2	ND	M	30	209.3
Hepatitis	None	6.2	3c	F	58	569
Hepatitis	None	6.8	3f	M	58	120.4
Kidney Trsp.	tac, MMF	6.1	3c	M	22	<0.4
Kidney Trsp.	tac, evero.	6.5	3c	M	57	<0.4
Kidney Trsp.	cyclosp, MMF	5.6	3f	M	77	607
Heart Trsp.	tac, evero, CT	7	3c	F	24	1440
Kidney Trsp.	tac, MMF	7.9	3c	M	73	5890 (Vir.)
Kidney Trsp.	tac, evero	7.5	3c	F	51	377

Notes:
Tac: tacrolimus; **Evero**: everolimus; **CT**: corticotherapy; **MMF**: mycophenolate mofetil; **Vir**: viremic (CMV DNA+).

2) Antibodies

Marker	Fluorochrome	clone	Provider
Pan-γδ	PE-Vio770	REA591	Miltenyi
CD3	Pacific Blue	HIT3a	BioLegend
Vδ1	APC-Vio770	REA173	Miltenyi
Vδ2	FITC	REA771	Miltenyi
Vγ9	PE-Cy5	A63663	Beckmann Coulter
IFN-γ	APC-AF700	4S.B3	Biolegend
CMV-IEp72	IgG1	6E1	Santa Cruz
Anti-mIgG	Goat Fab’2/AF546	N/A	Invitrogen

3) PCR primers

Specificity	Sense/antisense	Sequence (5' to 3')
HEV (ORF2/3region)	forward	GACAGAATTRATTTCGTCGGCTGG
HEV (ORF2/3region)	reverse	CCCTTRTCCTGCTGNGCATTCTCGACAGA
GAPDH	forward	GTCTCCTCTGACTTCAACAGCG
GAPDH	reverse	ACCACCCTGTTGCTGTAGCCAA

4) Supplemental figure 1

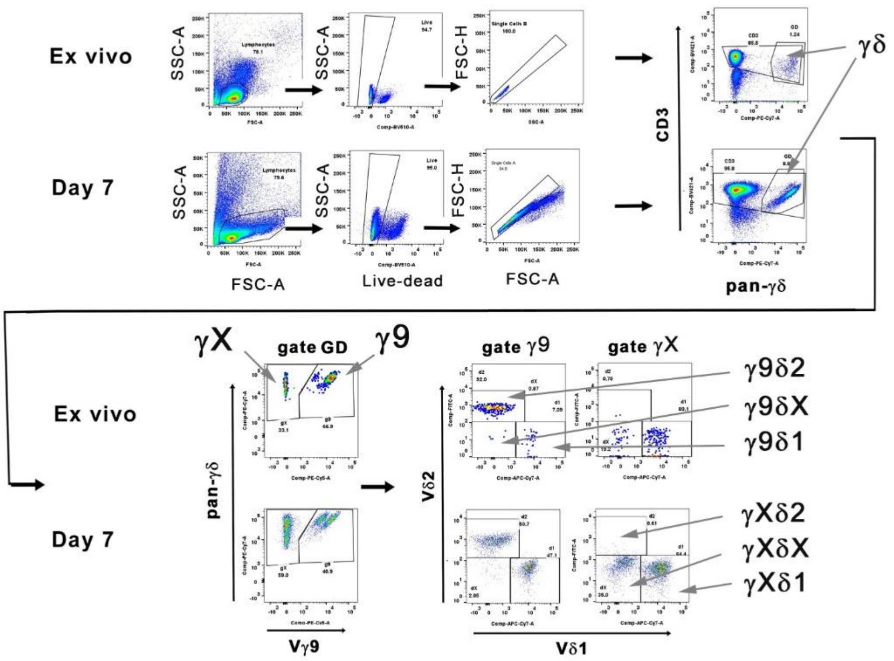

Gating Strategy for γδ T cell subset identification by flow cytometry

The gating strategies is shown for one representative individual. After gating on viable lymphocytes and exclusion of doublets, cells were first gated on the basis of Vγ9 expression and secondarily on δ1/δ2 expression.

5) Supplemental figure 2

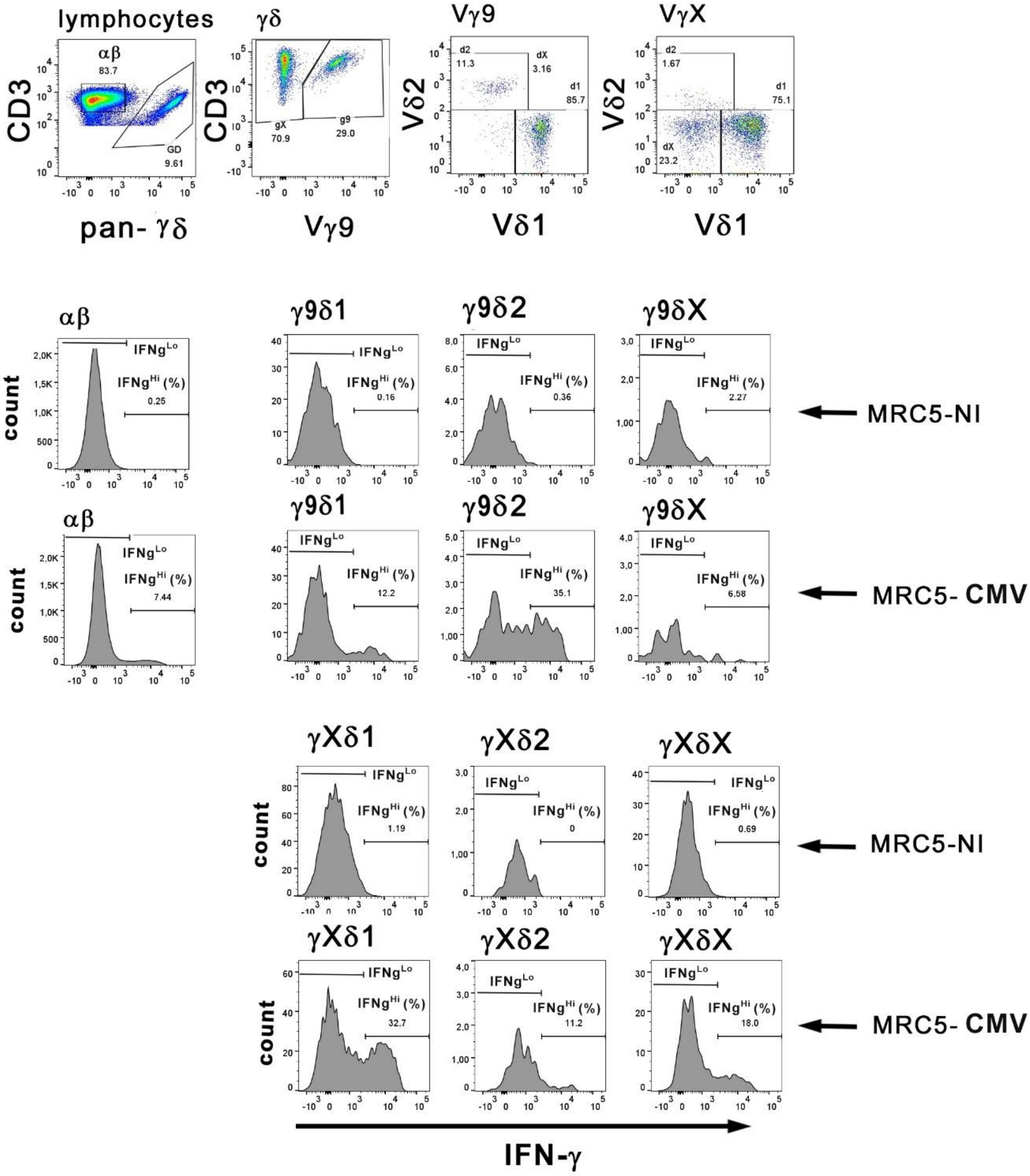

IFN-γ detection in lymphocyte subsets by flow cytometry

Representative histograms of intracellular IFN-γ expression in lymphocyte subsets gated as described in **Supp. Fig1**. IFN-γ response is shown for one representative individual. IL-2/IL-15-sensitized lymphocytes were recultivated 24hr with non-infected (NI) MRC-5 fibroblasts or fibroblasts infected with CMV in the presence of IL-2+IL-18.
